# Epicardial ablation of ventricular tachycardia in a patient with dilated cardiomyopathy due to Becker muscular dystrophy: a case report

**DOI:** 10.1093/ehjcr/ytaf406

**Published:** 2025-08-21

**Authors:** Irene Esteve-Ruiz, Maria Teresa Moraleda-Salas, Emilio Amigo-Otero, Javier Moreno, Pablo Morina-Vazquez

**Affiliations:** Arrhythmia Unit, Department of Cardiology, Hospital Juan Ramon Jimenez, Ronda Norte S/N, Huelva 21005, Spain; Arrhythmia Unit, Department of Cardiology, Hospital Juan Ramon Jimenez, Ronda Norte S/N, Huelva 21005, Spain; Arrhythmia Unit, Department of Cardiology, Hospital Juan Ramon Jimenez, Ronda Norte S/N, Huelva 21005, Spain; Cardiology Department, Hospital Ramón y Cajal, M-607, Km. 9, 100, Fuencarral-El Pardo, Madrid 28034, Spain; Arrhythmia Unit, Department of Cardiology, Hospital Juan Ramon Jimenez, Ronda Norte S/N, Huelva 21005, Spain

**Keywords:** Epicardial ablation, Ventricular tachycardia, Becker muscular dystrophy, Fractionated electrograms, Case report

## Abstract

**Background:**

Becker muscular dystrophy (BMD) is frequently associated with cardiac involvement. The underlying pathoanatomical substrate includes replacement of cardiomyocytes by fibrous tissue, leading to extensive myocardial fibrosis of the posterolateral wall of the left ventricular (LV) epicardium. Cardiac arrhythmias, including ventricular tachycardia (VT), are common in this condition, particularly when LV ejection fraction (LVEF) declines.

**Case summary:**

A 45-year-old male with dilated cardiomyopathy due to BMD presented for routine follow-up of his implantable cardioverter defibrillator (ICD). Device interrogation revealed multiple episodes of sustained VT, some terminated by antitachycardia pacing. Echocardiogram showed a mildly dilated LV with LVEF of 30%. In April 2024, he experienced an appropriate ICD shock for sustained VT, and substrate ablation was scheduled. Relying on predominant epicardial fibrosis known to BMD, a direct epicardial approach was performed and electroanatomical mapping (EAM) of the posterobasal LV revealed a large area of delayed, fractionated, and low-voltage electrograms (EGMs). Extensive ablation was performed with meticulous application near the atrioventricular annulus and left phrenic nerve region. Repeat EAM showed near-complete abolition of delayed potentials. No endocardial ablation was performed. Ventricular tachycardia remained non-inducible, and no sustained episodes or ICD shocks have been recorded during the 9-month follow-up.

**Discussion:**

Direct epicardial access may be the preferred ablation strategy for some cardiomyopathies such as BMD, where the arrhythmic substrate is epicardial. Detailed EAM with annotation of abnormal EGMs is crucial before ablation, and special care must be taken to avoid injury to critical structures such as the phrenic nerve or coronary arteries.

Learning points

- Becker muscular dystrophy often leads to cardiac involvement with predominant epicardial fibrosis, especially in the inferolateral wall of the left ventricle.
- A direct epicardial-only ablation approach may be appropriate in selected cases, especially when supported by imaging or literature and when institutional constraints limit the safety of endocardial access.
- Electroanatomical mapping is crucial to identify delayed and fractionated electrograms, which serve as effective ablation targets in epicardial ventricular tachycardia.

## Introduction

Becker muscular dystrophy (BMD) is an X-linked genetic disorder caused by mutations in the dystrophin gene, leading to progressive muscular weakness. It primarily affects proximal lower limbs, and it can be a rare cause of dilated cardiomyopathy (DCM), characterized by progressive heart failure (HF), conduction disorders, and frequent arrhythmic events.^1^ Ventricular tachycardia (VT) management in these patients can be challenging, and ablation is not often the first option of treatment due to the complex epicardial substrate.^2,3^

## Summary figure

**Figure:**
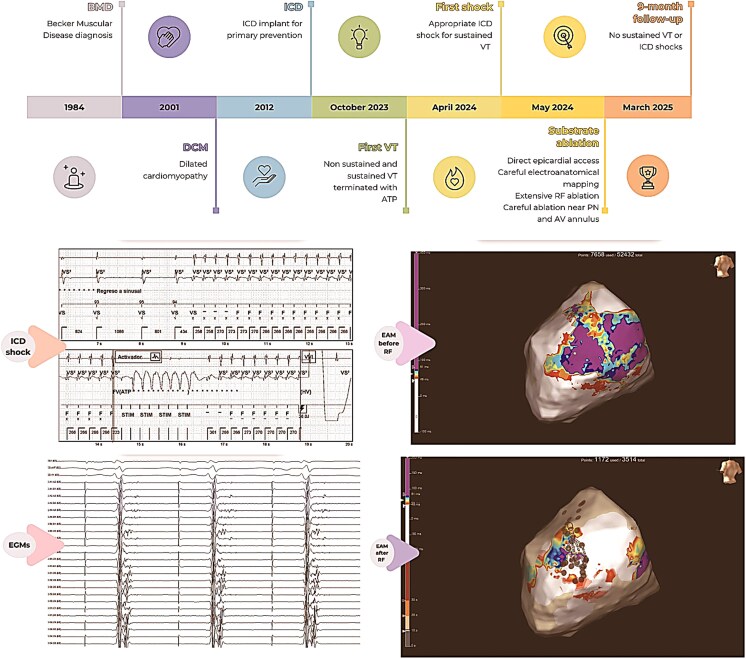


## Case presentation

We report a case of a 45-year-old male diagnosed with BMD in 1982 due to progressive muscle weakness. In 2001, he developed DCM, initiating treatment with beta-blockers and angiotensin-converting enzyme inhibitors. Nonetheless, his functional class worsened and left ventricular ejection fraction (LVEF) decreased to 30%, and in 2015, he underwent placement of an implantable cardioverter defibrillator (ICD).

In 2017, he developed atrial fibrillation, and treatment with amiodarone was initiated, remaining in sinus rhythm (SR). However, the treatment was discontinued due to the development of hyperthyroidism.

In 2023, he presented for his routine ICD follow-up, where episodes of sustained monomorphic VT, successfully treated with antitachycardia pacing, were detected. At that time, our patient was not receiving any antiarrhythmic drugs, and none were initiated. Basal electrocardiogram (ECG) revealed SR with normal atrioventricular (AV) conduction with non-specific intraventricular conduction defects, QRS 120 ms. Echocardiogram showed a mildly dilated left ventricle (LV) with LVEF 30%. Cardiac magnetic resonance (CMR) was performed in March 2024 but could not be interpreted due to significant artefacts from the ICD lead.

**Figure 1:**
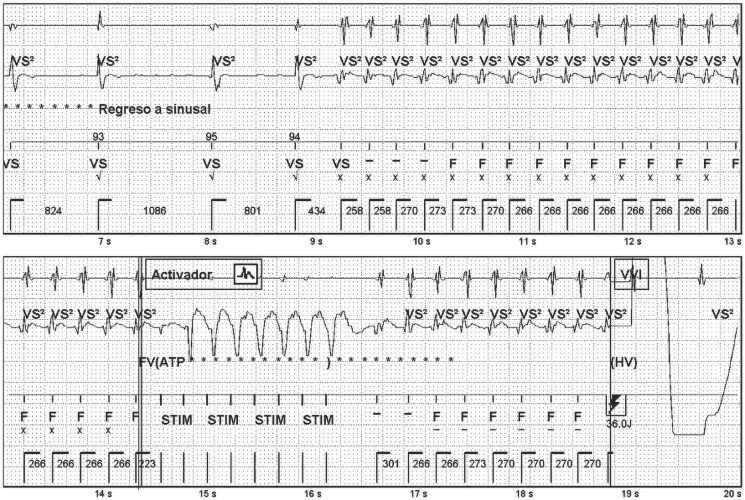
Sustained ventricular tachycardia treated with non-successful antitachycardia pacing during charge and appropriate shock.

In April 2024, he experienced an appropriate shock due to a fast, sustained VT (cycle length 266 ms), characterized by sudden onset and change in the intracardiac electrogram (EGM) (*Figure 1*). Substrate ablation of VT with electroanatomic mapping (EAM) system [Ensite X EP, St Jude Medical (SJM)] under general anaesthesia was scheduled. Based on the reviewed literature, a predominantly epicardial substrate was suspected. Consequently, a direct epicardial approach was chosen over initial endocardial mapping, to avoid initiating anticoagulation in a centre lacking onsite cardiac surgery.

An ultrasound-guided femoral puncture was performed and a decapolar catheter placed in the coronary sinus. Subxiphoid epicardial access was obtained using a 15 cm, 22-gauge needle introduced through an 18-gauge introducer needle, advancing a 0.038-inch diameter guidewire into the epicardial space. After radiologic confirmation of the guidewire’s position within the epicardium, the introducer set (Neff Percutaneous Access Set, Cook Medical) was advanced. An exchange guidewire was introduced, and a dual-steerable sheath (40 cm Agilis EPI, SJM) advanced over it, along with a mapping catheter (HD-Grid, SJM).

**Figure 2:**
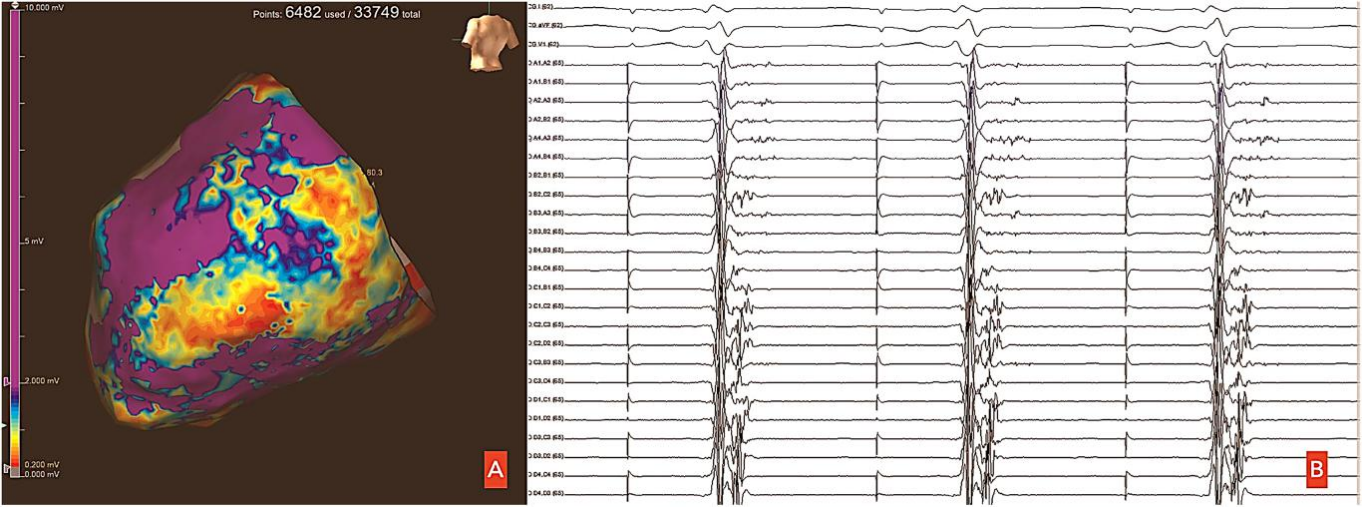
(*A*) Bipolar standard map. (*B*) Delayed and fractionated potentials found in the epicardial left ventricular inferolateral area during atrial pacing from the coronary sinus.

**Figure 3:**
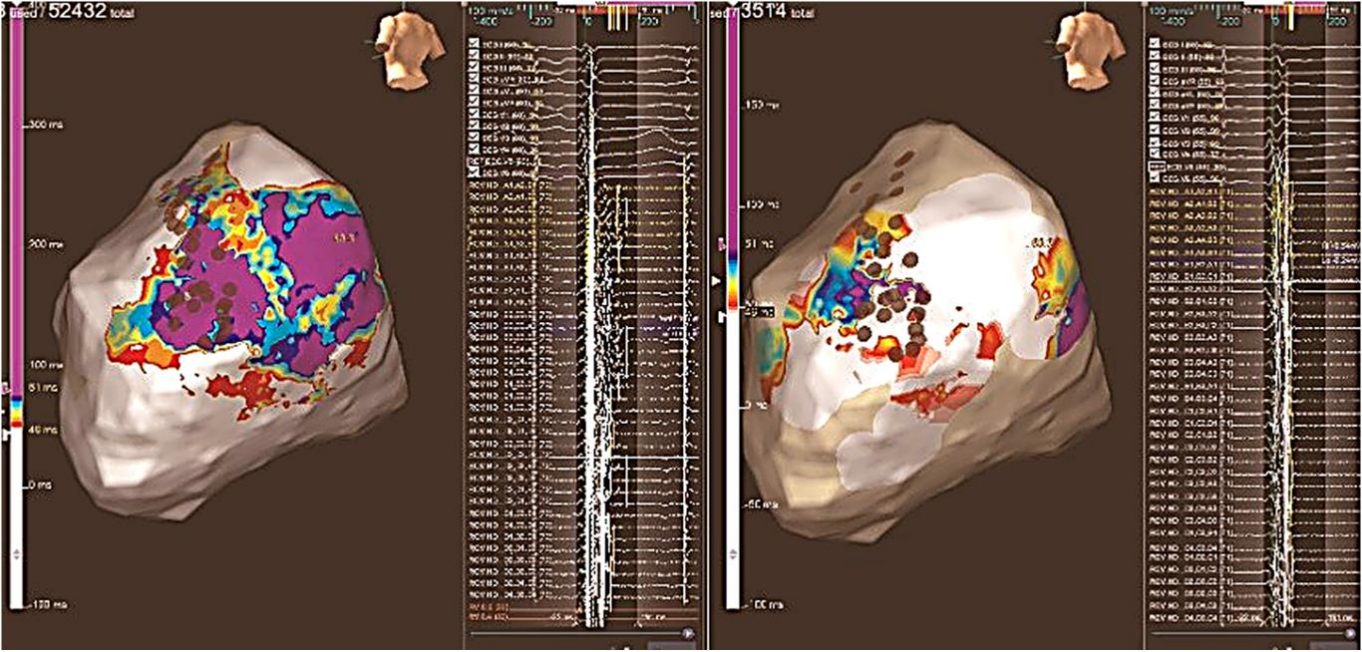
Change in late potential activation map of the epicardial left ventricular inferolateral area before and after ablation. Areas in white mark those areas where activation occurs during the QRS complex and areas in other colours where activation occurs after the QRS complex ends, with purple indicating the most delayed regions. Note the change in the delayed potentials after ablation, only remaining those near the atrioventricular annulus and the phrenic nerve innervation area.

**Figure 4:**
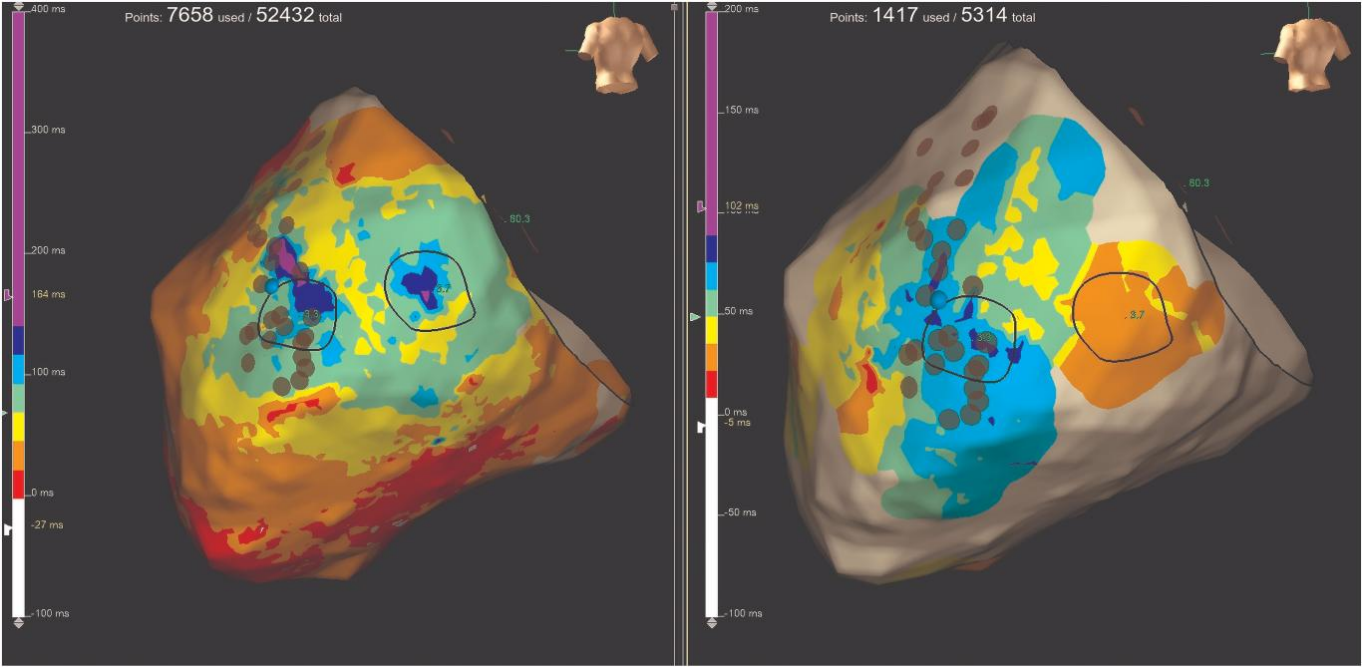
Isochronal late activation map of the epicardium before and after ablation. Note the isochronal crowding in the basal inferolateral wall, which resolves after ablation, while a small area of crowding persists in the phrenic nerve region due to the risk of permanent nerve injury.

During atrial stimulation from the coronary sinus, EAM of the epicardium was performed. A large area of low amplitude, fractionated, and delayed EGMs was found in the inferolateral wall of the LV (*Figure 2*), and a late potential activation map was generated (*Figure 3A*). Decrement-evoked potential mapping was performed, and identification of isochronal crowding was marked as potential deceleration zones (*Figure 4*). Decremental conduction was observed, as well as Wenckebach phenomenon reaching 2:1 conduction. Pacing from this area resulted in prolonged S-QRS intervals.

**Figure 5:**
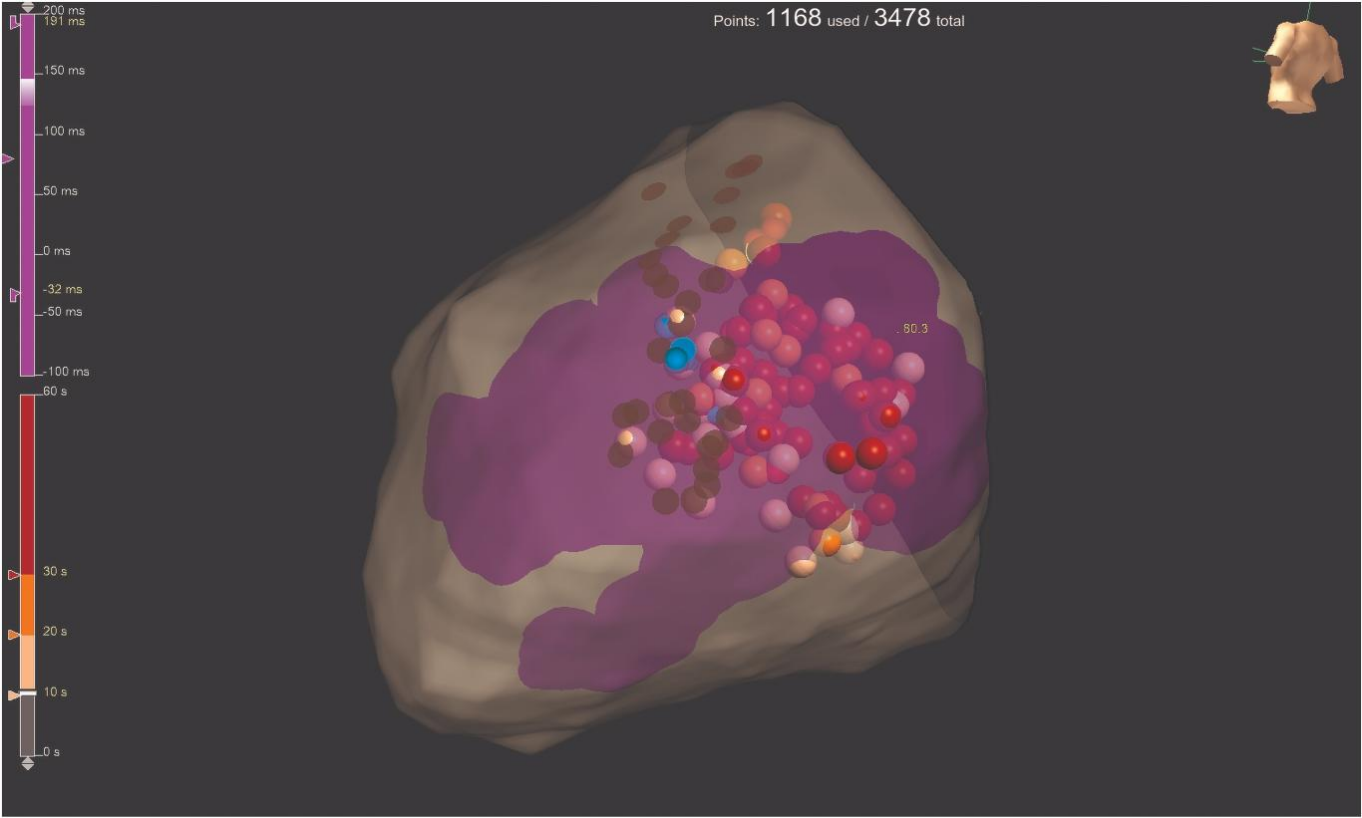
Radiofrequency ablation of target area. Orange, pink, and red dots mark radiofrequency applications; brown dots mark the region where phrenic nerve was locally captured from the epicardium and blue dots where phrenic nerve capture was temporarily lost during radiofrequency ablation.

Local capture of the left phrenic nerve (PN) from the epicardium was performed to delineate its anatomical distribution (*Figure 5*, brown dots). Additionally, coronary angiography was performed to delineate coronary arteries distribution.

Radiofrequency (RF) ablation using an irrigated ablation catheter (TactiFlex, SJM) was performed at 30–45 W to achieve adequate impedance drop (RF applications marked with orange, pink, and red dots, *Figure 5*). Abnormal EGMs, especially those exhibiting decremental conduction, were targeted for ablation. Phrenic nerve stimulation with high-output pacing was applied from the left subclavian vein during ablation in order to stop RF if PN capture was temporarily lost (blue dots, *Figure 5*). The area closest to the AV annulus was avoided.

Repeat EAM was performed, revealing near-complete abolition of delayed potentials, only remaining those near the AV annulus and the PN area (*Figure 3*). Programmed ventricular stimulation was performed without induction of any VT.

The patient was discharged after 3 days of hospitalization without any acute complications. No VT or ICD shocks have been registered in the 9-month follow-up. Our patient reported significant quality-of-life improvement, with resolution of pre-ablation activity limitations due to anxiety and fear of experiencing arrhythmic episodes.

## Discussion

Cardiac involvement in BMD is associated with DCM in at least one-third of cases.^4^ Cardiac arrhythmias frequently develop in this disease.^5^ Recent guidelines for the management of VT in these patients^6^ recommend an ICD implantation for scar-related VT; however, they fail to consider catheter ablation as a potential therapeutic option, highlighting a relevant gap regarding substrate-based strategies.

The underlying pathoanatomical substrate is characterized by cardiomyocyte replacement with fibrous tissue,^7^ with extensive myocardial fibrosis typically originating in the epicardium, as demonstrated in post-mortem studies,^8^ predominantly involving the posterobasal and lateral LV wall.^9^ Studies utilizing CMR with late gadolinium enhancement have confirmed these findings.^3,10^

Relying on predominant epicardial fibrosis known to BMD and that our center lacks onsite cardiac surgery, we avoided initial endocardial mapping, which would have required anticoagulation followed by epicardial puncture, an approach that we aimed to avoid. Therefore, we opted for epicardial access, reserving endocardial mapping only in case of normal epicardial mapping. Epicardial EAM revealed a large area of low amplitude, fractionated, and delayed EGMs, requiring extensive ablation and resulting in a prolonged procedure. Given the significative changes in the EGMs after ablation, we considered that additional endocardial mapping would not provide further benefit in this setting. Nonetheless, a combined endo-epicardial approach might have been a reasonable option, and it would be our strategy in case of future VT recurrences.

Limited data has been published in the literature on ablation for this substrate. To the best of our knowledge, only two prior cases of VT ablation in BMD have been published.^11,12^ In both cases, the substrate was entirely epicardial, with normal endocardial EAM and abnormal potentials found in the scar border, findings that are consistent with ours.

We chose not to induce VT before ablation as we lacked a 12-lead ECG of the clinical VT and haemodynamic compromise during VT mapping could be anticipated. Our ablation strategy was guided by electrophysiological findings, with detailed EAM and careful annotation of abnormal EGMs targeted for ablation, with particular emphasis on those exhibiting decremental conduction.

The ablation strategy focused on eliminating abnormal EGMs, which led to a long procedure. Special care was taken to avoid damage to sensitive structures near the target ablation area, including coronary arteries and PN. Epicardial ablation was accomplished without any complications. Repeat EAM showed a remarkable change, with near-complete abolition of delayed potentials found in the targeted area.

## Conclusion

This case highlights the potential value of a primary epicardial-only approach for VT ablation in patients with BMD or certain cardiomyopathies in which the arrhythmogenic substrate is located in the epicardium. Based on existing literature, we elected to forgo initial endocardial mapping to avoid anticoagulation-related risks in a center without onsite cardiac surgery. Detailed epicardial EAM revealed extensive abnormal EGMs targeted for ablation, which resulted in non-inducibility of VT, normalization of epicardial EGMs, and clinical improvement at 9 months. Our experience supports the consideration of a direct epicardial approach in similar clinical scenarios, particularly when anatomical, technical, and institutional factors converge.

## Lead author biography


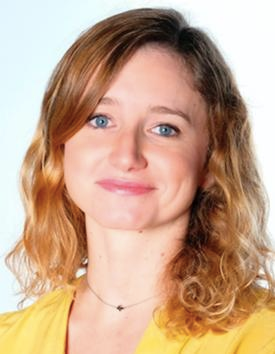


Doctor Irene Esteve-Ruiz is a cardiologist working as an EHRA-certified Cardiac Electrophysiology specialist in Spain. She graduated from Medical School at the University of Seville and completed her cardiology training at Valme University Hospital (Seville). She specialized in cardiac electrophysiology at Juan Ramón Jiménez Hospital (Huelva), where she currently works. She is also EHRA-certified in Cardiac Pacing and specializes in cardiac conduction system pacing (His bundle pacing and left bundle branch area pacing).

## Data Availability

The data underlying this article will be shared on reasonable request to the corresponding author.

## Notes

**Funding:** This work did not receive any specific grant from funding agencies in the public, commercial, or not-for-profit sectors.
